# Genome Sequences of Three Phytopathogenic Species of the Magnaporthaceae Family of Fungi

**DOI:** 10.1534/g3.115.020057

**Published:** 2015-09-28

**Authors:** Laura H. Okagaki, Cristiano C. Nunes, Joshua Sailsbery, Brent Clay, Doug Brown, Titus John, Yeonyee Oh, Nelson Young, Michael Fitzgerald, Brian J. Haas, Qiandong Zeng, Sarah Young, Xian Adiconis, Lin Fan, Joshua Z. Levin, Thomas K. Mitchell, Patricia A. Okubara, Mark L. Farman, Linda M. Kohn, Bruce Birren, Li-Jun Ma, Ralph A. Dean

**Affiliations:** * Center for Integrated Fungal Research, North Carolina State University, Raleigh, North Carolina 27606; † Department of Plant Pathology, North Carolina State University, Raleigh, North Carolina 27606; ‡ Department of Biochemistry and Molecular Biology, University of Massachusetts, Amherst, Massachusetts 01003; § The Broad Institute of MIT and Harvard, Cambridge, Massachusetts 02142; ** Department of Plant Pathology, Ohio State University, Columbus, Ohio 43210; †† USDA-ARS, Root Disease and Biological Control, Pullman, Washington 99164; ‡‡ Department of Plant Pathology, University of Kentucky, Lexington, Kentucky 40546; §§ Department of Biology, University of Toronto, Mississauga, Toronto, Ontario, L5L 1C6, Canada

**Keywords:** *Magnaporthe*, *Gaeumannomyces*, sequence, repetitive DNA, synteny

## Abstract

Magnaporthaceae is a family of ascomycetes that includes three fungi of great economic importance: *Magnaporthe oryzae*, *Gaeumannomyces graminis* var. *tritici*, and *Magnaporthe poae*. These three fungi cause widespread disease and loss in cereal and grass crops, including rice blast disease (*M. oryzae*), take-all disease in wheat and other grasses (*G. graminis*), and summer patch disease in turf grasses (*M. poae*). Here, we present the finished genome sequence for *M. oryzae* and draft sequences for *M. poae* and *G. graminis* var. *tritici*. We used multiple technologies to sequence and annotate the genomes of *M. oryzae*, *M. poae*, and *G. graminis* var. *tritici*. The *M. oryzae* genome is now finished to seven chromosomes whereas *M. poae* and *G. graminis* var. *tritici* are sequenced to 40.0× and 25.0× coverage respectively. Gene models were developed by the use of multiple computational techniques and further supported by RNAseq data. In addition, we performed preliminary analysis of genome architecture and repetitive element DNA.

Large-scale sequencing and bioinformatics-based genome analysis projects have broadened our understanding of fungal genome architecture, evolutionary relationships between species, and adaptation to environmental conditions. High-quality draft sequences of pathogenic fungal genomes can be platforms for studying genes that are involved in host−pathogen interactions, the infection cycle, and asexual propagation. Fungal genomes are often small but highly plastic, providing genetic diversity that is important in host or environmental adaptations but also contributing to divergence and speciation. Such plasticity can result in genome expansion and gene duplication. The gain, loss, and mutation of genes, particularly those involved in pathogenesis, have been attributed to repetitive elements in the genome, including retrotransposons and DNA transposons (Wöstemeyer and Kreibich 2002; Couch *et al.* 2005; Xue *et al.* 2012; Stukenbrock 2013). These data highlight the importance of quality genome sequences and genome-wide analysis to fungal researchers.

Plant fungal pathogens are a threat to a variety of crops worldwide. Among the most devastating, both economically and to global food security, are the Magnaporthaceae family of fungi, which contains several important plant pathogens including *Magnaporthe oryzae*, *Gaeumannomyces graminis* var. *tritici*, and *Magnaporthe poae. M. oryzae* is known as the rice blast fungus and primarily infects the leaf of its host plant, *Oryza sativa*, but can also infect other cultivated grasses like wheat and barley (Besi *et al.* 2001; Couch and Kohn 2002). Although it is difficult to calculate the specific monetary damage to crops caused by *M. oryzae*, conservative estimates suggest that 60 million of tons of rice have been destroyed in recent outbreaks (Zeigler *et al.* 1994; McBeath and McBeath 2010). *G. graminis* var. *tritici* is the causative agent of take-all disease in wheat. Unlike *M. oryzae*, which targets the leaf of the plant, *G. graminis* var. *tritici* attacks the roots of wheat plants, resulting in root rot. Hyphae of the soil-borne fungus wrap around the root and invade the root structure causing tissue necrosis. In acute infections, the pathogen can spread through the vascular system, causing loss of the head and subsequent killing of the plant (Besi *et al.* 2001; Freeman and Ward 2004). Similar to *G. graminis* var. *tritici*, *M. poae*, the causative agent of summer patch disease in turf grasses, attacks the roots of grasses causing root-rot and subsequent host-plant death (Besi *et al.* 2001).

Previous drafts of the *M. oryzae* genome have been published (as *Magnaporthe grisea*) with the whole-genome shotgun sequencing approach. The resulting draft genome had sevenfold sequencing coverage, and a subsequent analysis showed a family of G-protein coupled receptors that are unique to *M. oryzae* (Dean *et al.* 2005). Here, the genomes for *M. oryzae*, *M. poae*, and *G. graminis* var. *tritici* were sequenced with Sanger, Illumina sequencing of Fosmid vectors, and 454 next-generation sequencing technologies. The *M. oryzae* genome is finished to seven chromosomes, whereas *M. poae* and *G. graminis* var. *tritici* were sequenced to 40.0X and 25.0X coverage, respectively. In addition, we present a preliminary analysis of genome architecture and repetitive element content.

## Materials and Methods

### Genome sequencing

Sequencing of the Magnaporthaceae was performed through the Fungal Genome Initiative at the Broad Institute of Harvard and MIT (http://www.broadinstitute.org/). Sanger sequencing, 454 sequencing, and Illumina sequencing of Fosmid vectors were used for the Magnaporthaceae genomes. Both the *G. graminis* var. *tritici* and *M. poae* genomes were assembled by combining sequences generated with Sanger, Illumina, and 454 sequence technologies and assembled *de novo* with Newbler Assembly software (454 Life Sciences) using paired reads to identify contigs. A summary of sequencing can be found in Table 1.

**Table 1:** Sequencing project summary

Organism Name	*Magnaporthe oryzae*	*Magnaporthe poae*	*Gaeumannomyces graminis* var. *tritici*
Strain/isolate name	70-15	ATCC 64411	R3-111a-1
Assembly name	MG8	Mag_poae_ATCC_64411_V1	Gae_graminis_V2
Mitochondrial/plasmid assembly name(s)	MG7_MITO	Mag_poae_ATCC_64411_V1_Mito	Gae_graminis_V1_Mito
Sequencing platforms	Sanger	Sanger/454/ABI	Sanger/454/ABI
Sequencing coverage	Finished to seven chromosomes	40.0×	25.0×
Genbank accession	AACU03000000	ADBL01000000	ADBI00000000
Gene numbering	MGG_#####	MAPG_#####	GGTG_#####
NCBI project ID	13840	37933	37931

Sequencing was performed at the Broad Institute as part of the Fungal Genome Initiative. NCBI, National Center for Biotechnology Information.

The Sanger-based *M. oryzae* genome (Dean *et al.* 2005) was finished by combining a semiautomated and manual finishing pipeline at the Broad Institute and was deposited at the National Center for Biotechnology Information (NCBI) with the accession number of AACU00000000.3. Significant retrotransposon content led to a compromised *M. oryzae* genome sequence. To finish the genome sequence, unique sequence anchors were verified manually. Contigs and scaffolds were extended by manual placement of plasmid and Fosmid vector end sequences. The remaining gaps were filled by searching unique contig end sequences against unincorporated reads. *In vitro* transposition also was used to determine the entire sequence of plasmid (4 kb average insert size) and Fosmid clones (40 kb average insert size). An optical physical map served as an important mechanism for confirmation of added sequence. The optical map facilitated arrangement of the final scaffolds into pseudochromosomes. Telomere sequence was improved through the use of telomere Fosmid sequence (Farman and Kim 2005; Starnes *et al.* 2012). These data allowed for recruitment in additional unused whole-genome sequence reads. Final quality control of the sequence involved review of optical map anomalies, Fosmid clone mate-pair violations, and a list of missing genes compared with the draft sequence.

Sites of misassembly in *M. oryzae* were recognized by the presence of inappropriately placed reads and read pairs, along with discrepancies with the optical map. Misassemblies were removed by breaking the existing assembly at discrepant sites. A core set of Fosmid clones was identified from problem areas that had both of the end reads reliably placed in the genome assembly. The assembly was manually extended from these high-confidence anchors using both preexisting sequence data (primarily Fosmid end sequence pairs) and from newly generated sequence generated by walking using custom primers, as well as by transposing Fosmid and plasmid clones. As an independent check on the manually extended sequence, we correlated the sequence with the optical and physical maps. The sequence of the Fosmids that had been previously identified as containing telomeric repeats and sequenced was incorporated into the assembly (Farman and Kim 2005; Rehmeyer *et al.* 2006). The draft consensus sequence was used to recruit additional Broad shotgun data by sequence identity and read mate pairs. Finally, the positions of all Fosmid mate pairs were examined across the final consensus sequence. Mate-pair violations were investigated and corrected when necessary.

### Gene annotation

Gene annotation was performed by the Broad Institute using previously published annotation (Dean *et al.* 2005) and incorporated expression data generated by sequencing RNA libraries (outlined in *RNAseq*). In summary, the GenBank nr database (http://www.ncbi.nlm.nih.gov/) was used in a Blast similarity search for putative Magnaporthaceae genes. Blast hits with an e-value of 1e-10 were used as evidence for gene prediction. Hmmer analysis (http://hmmer.janelia.org/) was used to further identify homologs in the target genomes with the pFAM protein domain library. Finally, expressed sequence tags (ESTs) were aligned to the genome with BLAT (https://genome.ucsc.edu/cgi-bin/hgBlat). Alignments with 90% identity over 50% of the length of the EST were considered valid. Gene models were built with EST clusters and the FindEstOrf tool through the Broad Institute. Computational gene models were produced as previously described (Dean *et al.* 2005) by using a number of gene-prediction tools including GeneMark (Borodovsky and McIninch 1993), GENEID (Blanco *et al.* 2007), FGENESH (Softberry, Mount Kisco, NY), and EST computational and manual modeling.

### RNAseq

*M. oryzae* strain 70-15 and *M. poae* ATCC 64411 RNA were extracted from a subset of nine growth conditions for RNAseq analysis as previously described (Nunes *et al.* 2011): cold (4°), heat (42°), salt (500 mM NaCl), light, dark, melanizing, potato dextrose broth (1× PDB, Fisher Scientific, Waltham, MA), V8 juice medium (10% v/v), or complete medium (CM, Weiland 2004). The strains were grown in liquid CM, V8 broth, or PDB at 25° at 200 rpm for 3 d. Heat and cold treatments were performed by submerging CM culture flasks in water baths at 4° or 42° for 15 min before RNA extraction. For NaCl treatment, NaCl was added to a final concentration of 500 mM for 15 min before RNA extraction. Additionally, mycelia were grown for 3 d in CM in the absence or presence of light (dark or light condition). For melanizing conditions, mycelia were grown in the presence of light for 4 d before RNA harvest. Mycelia were harvested, washed with sterile water, blot dried, and RNA was extracted from the fresh tissue. RNA extraction was performed as described previously (Gowda *et al.* 2006; Nunes *et al.* 2011). RNA samples were treated twice with DNAseI to ensure they were free of DNA contamination. RNA from three separate mycelial preparations for each growth/treatment condition were pooled before library construction for RNA sequencing.

For *M. oryzae* samples, polyA^+^ RNA was isolated by using two rounds of selection with the Dynabeads mRNA Purification Kit (Life Technologies, Carlsbad, CA) starting from 50 μg of total RNA. All of the polyA^+^ RNA was used for construction of dUTP second-strand marking libraries as previously described (Levin *et al.* 2010), except that RNA was fragmented in 1× RNA fragmentation buffer (Affymetrix, Santa Clara, CA) for 4 min at 80° and after first-strand cDNA synthesis a 1.8× RNAClean SPRI beads (Beckman Coulter Genomics) cleanup was used instead of phenol:chloroform:isoamyl alcohol (25:24:1) extraction and ethanol precipitation. For *M. poae* samples, polyA^+^ RNA was isolated by using three rounds of selection with the Dynabeads mRNA Purification Kit (Life Technologies) starting from 75 μg of total RNA. Then, 200 ng of polyA^+^ RNA was used for construction of dUTP second-strand marking libraries as previously described, except RNA was fragmented as for *M. oryzae*. RNAseq libraries were subjected to paired-end deep sequencing using GAII Illumina technology (Illumina, Inc., San Diego, CA).

*G. graminis var. tritici* isolate R3-111-1a 1B was cultured in 1× PDB or 1/3× PDB for 5 d at 23°. Mycelia were washed four times in autoclaved nanopure water over filter paper under gentle vacuum, frozen in liquid nitrogen, and stored at −80°. Five-day-old mycelia grown in 1× PDB were also treated at 40° or 2° in 1× PDB, or at 22° in 1× PDB containing 0.5 M NaCl for 1 hr before harvest. Total RNA was obtained using TRIzol reagent (Invitrogen, Carlsbad, CA) and isopropanol/citrate (Okubara *et al.* 2010). RNA quality was visualized on 1% formaldehyde agarose gels. Fifty-microgram aliquots of RNA were treated with DNAse (Turbo DNA-free kit, Ambion, Inc., Austin, TX) and passed through RNeasy columns (QIAGEN, Inc., Valencia, CA). DNase-treated RNA did not produce an actin PCR product when amplified with *M. oryzae* primers Actin F and Actin R (Gowda *et al.* 2010) designed to produce an actin PCR product only if an intron was present.

Following pooling of RNA from three separate mycelial preparations for each growth/treatment, libraries were constructed similarly to *M. orzyae* samples, except that 29 to 36 micrograms of total RNA were used as input and an additional two rounds of polyA^+^ selection was needed for three (40°; 2°; and 0.5 M NaCl) samples.

RNAseq transcript reads were aligned to their respective reference genomes by using TopHat and Bowtie software (Langmead 2010; Kim and Salzberg 2011) and Inchworm RNAseq assembly software (http://trinityrnaseq.github.io/). PASA was used for cDNA-based genome annotation (Haas *et al.* 2003). Together, these algorithms were used to define introns, exons, untranslated regions, and alternative splicing isoforms of transcripts. A summary of RNAseq read data can be found in Table 2.

**Table 2:** RNAseq reads per treatment

Biological Treatment	*M. oryzae*	*M. poae*	*G. graminis*
2°	−	−	38,264,704
4°	41,670,516	55,296,984	−
40°	−	−	52,913,434
42°	47,383,418	51,115,288	−
NaCl (500 mM)	51,560,152	49,951,750	46,568,806
Light	43,071,966	−	−
Dark	46,564,826	54,886,478	−
Melanized	−	44,351,958	−
1× PDB	−	−	30,717,700
V8 medium	−	−	31,607,460
Complete Medium	−	108,842,14	−

Magnaporthaceae species were grown in complete medium before being subjected to different conditions for 15−60 min (2°, 4°, 40°, 42°, NaCl), 3-5 d (light, dark, 1× PDB, V8 medium, complete medium, and melanizing) before RNA extraction. RNA libraries were subjected to paired-end deep sequencing using GAII Illumina technology. RNAseq read were assembled and aligned to their respective genomes using Bowtie, TopHat, and Inchworm software. PDB, potato dextrose broth.

### Genome architecture and repetitive element analysis

Syntenic regions were found using CoGe Synmap (dotplots) and GEvo (https://www.genomevolution.org/coge/). The number of syntenic blocks was based on the GEvo output file after Synmap analysis, and the amount of syntenic DNA was calculated by using the start and stop positions for each syntenic block. Repetitive element analysis was performed by using the RepeatModeler and RepeatMasker programs (http://www.repeatmasker.org). To summarize, *de novo* repetitive element libraries were created with RepeatModeler using the RMBlast NCBI search engine. Final classified consensus files for *M. poae* and *G. graminis* var. *tritici* were used as libraries for subsequent repetitive element searches. Similar repetitive elements were aligned by RepeatModeler and collapsed into their parent families. Repetitive element families were classified by RepeatModeler. All sequences were analyzed by BlastX (http://blast.ncbi.nlm.nih.gov/Blast.cgi) against the nonredundant protein sequence database to identify any known retrotransposon or DNA transposon proteins. Further confirmation of library sequences was performed using the EMBOSS suite of bioinformatics tools (http://emboss.bioinformatics.nl/). Long terminal repeats were identified using POLYDOT whereas terminal inverted repeats were identified using EINVERTED. RepeatMasker was used to identify the locations of repetitive elements.

### Data availability

Genome sequences, transcript sequences, genome statistics, and annotation are available for download via Genbank (see Table 1 for accession numbers). RepeatModeler libraries are available in the supplemental materials for *M. oryzae* (supporting information, File S1), *M. poae* (File S2), and *G. graminis* var. *tritici* (File S3).

## Results

### Whole-genome sequencing

The *M. oryzae* genome was finished to seven chromosomes with the exception of ∼530-kbp segment (scaffold 8) that could not be robustly assigned to a particular chromosome, whereas 40-fold and 25-fold coverage was achieved for *M. poae* and *G. graminis* var. *tritici* draft genomes, respectively (Table 1). The *M. oryzae* genome sequence consisted of 219 contigs assembled into eight scaffolds, with a total genome size of 41.0 Mbp (Table 3). *M. poae* has the smallest genome at 39.5 Mbp, which consisted of 3106 contigs assembled into 205 scaffolds. The *G. graminis* var. *tritici* genome was the largest at 43.6 Mbp and was made up of 1808 contigs assembled into 513 scaffolds. Total genomic G+C content was similar between *M. poae* and *G. graminis* var. *tritici* at just under 57%. In contrast, the G+C content of *M. oryzae* was lower at approximately 52%. The G+C content of both *M. poae* and *G. graminis* var. *tritici* are high for fungal species, which range from ∼30% to ∼57% (Jung *et al.* 2008); however, the biological repercussions are unclear (Li and Du 2014).

**Table 3:** Genome statistics

Organism Name	*M. oryzae*	*M. poae*	*G. graminis var. tritici*
Genome size, bp	41,027,733	39,503,331	43,618,147
Contig N50, bp	823,590	16,565	48,943
Scaffold N50, bp	6,606,598	3,426,601	6,703,616
Contig count	219	3,106	1,808
Scaffold count	8	205	513
Protein-coding genes	12,696	12,113	14,255
Coding regions in the genome, %	61.34	63.59	63.38
Gene length median, bp	1,755	1,823	1,711
mRNA length median, bp	1,556	1,584	1,494
CDS length median, bp	1,083	987	1,041
Exon length median, bp	374	412	370
Intron length median, bp	88	87	88
Exon per spliced transcript	3.22	3.3	3.21
5-UTR length median, bp	267	301	246
3-UTR length median, bp	298	343	304
Intergenic region length median, bp	742	654	580
Contig gap length median, bp	100	537	481
Genome G+C content, %	51.61	56.99	56.85
Genic region G+C content, %	54.09	58.77	59.8
Intergenic region G+C content, %	47.66	52.57	50.77
mRNA G+C content, %	55	59.31	60.67
CDS G+C content, %	57.63	61.65	62.76
Exon G+C content, %	55	59.31	60.67
Intron G+C content, %	46.58	53.76	53.03
5-UTR G+C content, %	48.8	56.29	56.97
3-UTR G+C content, %	46.4	51.75	51.91
Genes with 5-UTR, %	8,120	8,017	8,590
Genes with 3-UTR	8,153	7,749	8,802
Spliced genes	10,341	9,388	11,176
Average exons per transcript	2.78	2.76	2.71
Alternatively spliced genes	796	877	885
rRNA genes	40	26	13
tRNA genes	325	167	273

Magnaporthaceae species were sequenced using Sanger sequencing (*M. oryzae*), or Sanger sequencing, ABI, and 454 Next-Generation sequencing (*M. poae* and *G. graminis* var. *tritici*). *M. oryzae* was finished to seven chromosomes, whereas *M. poae* and *G. graminis* var. *tritici* were sequenced to 40-fold and 25-fold coverage, respectively. mRNA, messenger RNA; CDS, coding sequence; UTR, untranslated region; rRNA, ribosomal RNA; tRNA, transfer RNA.

Multiple computational methods were used to create gene models. RNAseq alignments were used to predict transcript introns, exons, untranslated regions, and alternative splicing isoforms (Table 2 and Table 3). The *M. oryzae* and *M. poae* genomes have a similar number of protein-coding genes, whereas that of *G. graminis* var. *tritici* had approximately 2000 more protein-coding genes (Table 3). However, the proportion of each genome that is represented by protein-coding gene sequences is similar among the three, ranging from 61.3 to 63.6% of each genome. mRNA median length, the number of exons spliced per transcript, and the number of introns per transcript were similar among all three species. The number of spliced genes varied, with *M. poae* showing the lowest number at 9388 and *G. graminis* var. *tritici* with the highest at 11,176. *G. graminis* var. *tritici* and *M. poae* showed similar numbers of alternatively spliced genes at 885 and 877, respectively, whereas *M. oryzae* had fewer spliced genes with 796 found after RNAseq data analysis. Although these data show that there are clear differences in gene number and splicing between the three species, additional analysis is necessary to understand how these differences affect cell processes and pathogenesis.

### Repetitive element analysis

Inverted repeats and transposon DNA sequences have been found in a variety of fungal plant and animal pathogens, including the wheat pathogen *Mycosphaerella graminicola* (Dhillon *et al.* 2014) and the human pathogen *Cryptococcus neoformans* (Idnurm *et al.* 2005). It has been hypothesized that repetitive elements may contribute to speciation and divergence even among closely related species of fungi (reviewed in Wöstemeyer and Kreibich 2002; Raffaele and Kamoun 2012; Stukenbrock 2013). Repetitive elements can be divided into two classes: class I retrotransposons and class II DNA transposons. Class I uses an RNA intermediate to copy and paste itself into new sites in the genome, whereas class II uses a cut and paste mechanism to excise themselves from the genome and insert in new locations. Previously, the repetitive element content of the *M. oryzae* genome was described (Dean *et al.* 2005; Xue *et al.* 2012); however, repetitive element analysis had not been performed on *M. poae* or *G. graminis* var. *tritici*.

We used RepeatModeler, which uses RepeatScout (Price *et al.* 2005) and RECON (Bao and Eddy 2003) *de novo* repeat library algorithms, and RepeatMasker to identify and classify the repetitive elements in the *M. poae* and *G. graminis* var. *tritici* genomes. Families of repetitive elements found by RepeatModeler were confirmed by BlastX against the NCBI nonredundant protein database and alignment against previously identified *M. oryzae* repetitive elements (Dean *et al.* 2005). The *M. oryzae* genome contained the greatest proportion of repetitive DNA sequence at 10.13% (Table 4), which is consistent with previous reports (Dean *et al.* 2005; Xue *et al.* 2012). Repetitive element content in *M. oryzae* primarily consisted of retrotransposon sequences, which accounted for more than 57%. Similar to *M. oryzae*, *G. graminis* var. *tritici* repetitive element content was more than 63% retrotransposon sequences. The total proportion of the genome was less than that of *M. oryzae*, at 6.71% repetitive DNA. In contrast, the repetitive content of *M. poae* was a small proportion of the genome, at 1.1%. DNA transposon sequences represented the largest proportion of the repetitive content of *M. poae* at just over 40%; retrotransposon elements and unknown/unclassified elements were significant at 32.83% and 20.26%, respectively. These data suggest that although *G. graminis* var. *tritici* has a larger genome than do *M. oryzae* or *M. poae*, it is not due to repetitive element DNA but likely due to increased numbers of paralogs and novel genes compared to the other two species.

**Table 4:** Repetitive elements in the Magnaporthaceae

	*M. oryzae*			*M. poae*			*G. graminis var. tritici*		
	Total Length, bp	Repetitive Content, %	Genome, %	Total Length, bp	Repetitive Content, %	Genome, %	Total Length, bp	Repetitive Content, %	Genome, %
Class I (retrotransposon)									
LTR/Gypsy	1790041	43.08	4.36	142221	32.83	0.36	1317401	44.99	3.02
LTR/Copia	375743	9.04	0.92				529308	18.08	1.21
Unknown	212069	5.10	0.52						
Subtotal	2377853	57.23	5.80	142221	32.83	0.36	1846709	63.07	4.23
Non-LTR retrotransposons									
LINE/Tad1	690463	16.62	1.68				342293	11.69	0.78
Unknown	135550	3.26	0.33	176681	40.78	0.45	13605	0.46	0.03
* *Subtotal	826013	19.88	2.01	176681	40.78	0.45	355898	12.16	0.82
Class II (DNA tranpsosons)									
DNA/TcMar-Fot1	514336	12.38	1.25	19406	4.48	0.05	157841	5.39	0.36
DNA/PIF-Harbinger							18302	0.63	0.04
DNA/hAT-Ac							17124	0.58	0.04
Unknown	306017	7.36	0.75	7139	1.65	0.02			
Subtotal	820353	19.74	2.00	26545	6.13	0.07	193267	6.60	0.44
Other									
Unknown	131047	3.15	0.32	87794	20.26	0.22	532034	18.17	1.22
Total	4155266		10.13	433241		1.10	2927908		6.71

Repetitive elements were collapsed into parent families and classified by RECON. BLASTx was used to confirm classification. Unclassified families were further analyzed for TIRs and LTRs using EINVERTED and POLYDOT, respectively. LTRs, Long terminal repeats; TIRs, terminal inverted repeats.

### Genome synteny

**Figure 1:**
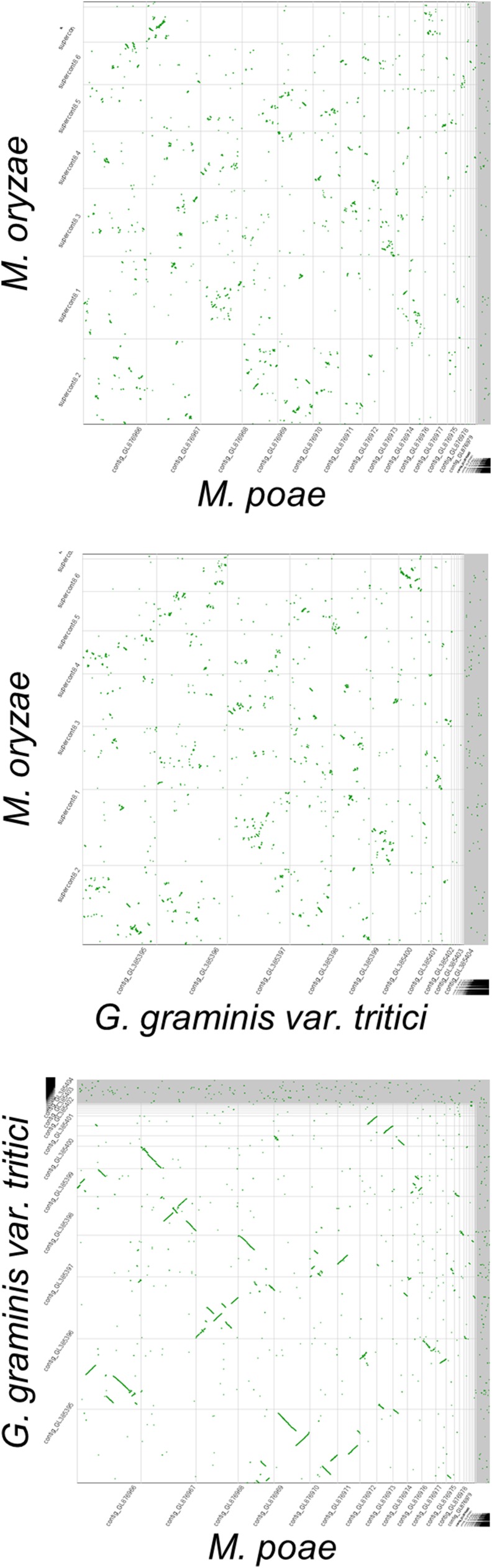
Genome synteny. CoGe genome synteny analysis software was used to compare the genomes of *M. oryzae* and *M. poae* (top), *M. oryzae* and *G. graminis* var. *tritici* (middle), and *G. graminis* var. *tritici* and *M. poae* (bottom). Regions of synteny are plotted as green dots. Highly syntenic regions appear as linear segments when plotted.

The conservation of genetic loci (synteny) can be used to examine the evolutionary relationships between species. We previously reported that there was little synteny conserved between *M. oryzae* and the closely related *Neurospora crassa*, suggesting that the genome of *M. oryzae* may be highly plastic (Dean *et al.* 2005). Here we compared the genomes of the three Magnaporthaceae using CoGe Synmap software to identify regions of synteny (Figure 1). Analysis of CoGe Synmap outputs revealed that the genomes of *M. poae* and *G. graminis* var. *tritici* share 34,063 syntenic blocks, which accounted for approximately 19.1 Mbp of sequence. In contrast, *M. oryzae* shared fewer regions of synteny with *M. poae* (19,322 blocks, 7.2 Mbp) and *G. graminis* var. *tritici* (21,076 blocks, 8.4 Mbp). These data support previous evidence that, despite the difference in genome size between *M. poae* and *G. graminis* var. *tritici*, they diverged more recently than *M. oryzae* (Zhang *et al.* 2011).

## Discussion

The Magnaporthaceae family of fungi is of both economic and social importance. Rice blast disease affects one of the largest food crops in the world and results in the loss of millions of tons of food. More recently, *M. oryzae* has become a model to study fungal plant pathogens. Thus, the importance of high-quality genome sequencing and gene annotation is a critical tool for the research community. Here we provide the finished sequence of *M. oryzae* as well as 40.0× and 25.0× coverage draft sequences of the related species *M. poae* and *G. graminis* var. *tritici*, respectively.

Assembly and annotation of the genomes was performed in association with the Broad Institute’s Fungal Genome Initiative. Multiple methods were used to produce computational gene models including the use of ESTs, homologous gene searches, and Blast searches. Putative gene models were aligned to RNAseq transcript data that were produced under a variety of conditions to further support the gene models. Together, these techniques provide researchers with high-confidence annotation and gene models for use in future analysis and experimentation on the Magnaporthaceae.

Initial genome architecture was examined by using both repetitive element analysis and genome synteny. Similar to previous studies, our data show that approximately 10% of the *M. oryzae* genome consists of repetitive elements (Dean *et al.* 2005; Xue *et al.* 2012). The majority of the repetitive content in both *M. oryzae* and *G. graminis* var. *tritici* was made up of retrotransposon sequence. Retrotransposons use the mechanism of “copy and paste” to propagate, allowing for many copies to be inserted throughout the genome. Thus, it is unsurprising that retrotransposons make up the majority of repetitive content in these two species. These data are similar to those found in other fungi, including the rice endophyte *Harpophora oryzae* (Xu *et al.* 2014), the human pathogens *Sporothrix schenckii* and *Sporothrix brasiliensis* (Teixeira *et al.* 2014), the corn leaf blight disease–causing *Cochliobolus heterostrophus* (Santana *et al.* 2014), where the composition of repetitive elements is primarily made up of retrotransposons. However, despite the larger genome, only 7% of the *G. graminis* var. *tritici* genome was made up of repetitive element sequence. Additional analysis of gene copy number and tandem repeats may shed light on the nature of the larger genome.

The genome of *M. poae* had the lowest amount repetitive content, at just over 1%. These results may be due to the loss of repetitive element sequences during assembly of the genome. Thus, repetitive element content analyses may need to be revisited with higher coverage sequencing, longer read sequencing, or a finished genome sequence of *M. poae*.

Synteny is the conservation of gene loci across species. Comparison of genomes and identification of syntenic regions can shed light on important gene linkages as well as evolutionary relationships between species. Here, we compared the genomes of *M. oryzae*, *M. poae*, and *G. graminis* var. *tritici* and looked for shared syntenic regions. We found that *M. oryzae* was most divergent, showing fewer regions of synteny compared with *M. poae* and *G. graminis* var. *tritici*. Thus, synteny suggests that *M. poae* and *G. graminis* are more closely related to each other than either are to *M. oryzae*. These data further support data previously published by Zhang *et al.* (2011). In contrast to *M. oryzae*, which primarily infects the host plant’s leaves, both *M. poae* and *G. graminis* var. *tritici* infect the roots and crown of their host plants. Interestingly, the genomes of *M. poae* and *G. graminis* var. *tritici*, despite their shared regions of synteny, have the greatest difference in size at approximately 4Mbp. Analysis of orthologous and paralogous genes between the two species may provide insight into their shared routes of pathogenesis compared with *M. oryzae*.

## Supplementary Material

Supporting Information
