# Supplementary material for: Genome Sequences of Three Phytopathogenic Species of the Magnaporthaceae Family of Fungi
Source: G3 (Bethesda). 2015 Sep 28;5(12):2539–45. doi: 10.1534/g3.115.020057 (PMC4683626; doi:10.1534/g3.115.020057)
Supplement: Supporting Information [file supp_5_12_2539__index.html]

Genome Sequences of Three Phytopathogenic Species of the Magnaporthaceae Family of Fungi — Supporting Information 

# Genome Sequences of Three Phytopathogenic Species of the Magnaporthaceae Family of Fungi

## Supporting Information for Okagaki *et al.*, 2015

**Files in this Data Supplement:**

- File S1 - *De novo* repetitive element library for *M. oryzae*. (.txt, 112 KB)
- File S2 - *De novo* repetitive element library for *M. poae*. (.txt, 29 KB)
- File S3 - *De novo* repetitive element library for *G. graminis* var. *tritici*. (.txt, 138 KB)
